# Support for Children of Parents With Mental Illness: An Analysis of Patients’ Health Records

**DOI:** 10.3389/fpsyt.2022.778236

**Published:** 2022-03-09

**Authors:** Kjersti Bergum Kristensen, Camilla Lauritzen, Charlotte Reedtz

**Affiliations:** Regional Center for Child and Adolescent Mental Health and Child Welfare, Faculty of Health Sciences, UiT The Arctic University of Norway, Tromsø, Norway

**Keywords:** children of parents with mental illness, parental mental illness, mental healthcare services for adults, healthcare professionals, preventive intervention, support of patients’ children

## Abstract

**Introduction:**

Children of parents with a mental illness (COPMI) are at risk of behavioral, emotional, and cognitive difficulties and diagnoses. Support and information about parents’ mental illness may contribute to improve their lives, which is the purpose of the intervention Child Talks (CT). This study aimed to investigate the participation rate of CT, characteristics of participating patients and children, and themes in sessions with children.

**Materials and Methods:**

Data were collected from 424 electronic patient journals written by healthcare professionals (H) for patients admitted to a clinic for mental health and substance use disorders in the years 2010–2015. Both quantitative statistical analysis and qualitative thematic analysis were carried out.

**Results:**

Eighteen percent of assessed parents with minor children received the CT intervention and children participated in half of them. Participating children more often knew about their parent’s treatment and condition when initially assessed, and more often lived with the hospitalized parent. Three main themes were identified in sessions with children; communication about parental mental illness within the family, childrens’ struggles, and healthcare professionals’ (HCPs) evaluation of the child’s situation and need for further support.

**Discussion:**

Sessions with patients’ children appeared to be relatively rare, and participating children did not necessarily receive appropriate information, support, or follow-up. To ensure that HCPs provide quality support and follow-up to COPMI, the routines and the training of HCPs need to be improved.

## Introduction

In Norway, 12.2% of children have parents who are receiving treatment for mental illness and/or alcohol use disorder each year (1). According to Norwegian (2), and international estimates (3) approximately one third of patients in adult psychiatric services are parents of minor children. Children of parents with a mental illness (COPMI) have an elevated risk of developing emotional, behavioral, and cognitive difficulties that can result in poorer life outcomes regarding educational level, ability to work, socioeconomic status, and ability to establish meaningful relationships with others (4–8). COPMI are at risk of developing the same illness as their parents, as well as other mental illnesses (9). Half of the children of parents with a severe mental illness (SMI) are at risk of developing a mental illness themselves by the age of 20, and one third are at risk of developing a SMI (10).

The transmission of mental illness from one generation to the next is a complex process. Such transmission is influenced by the interaction of factors related to the mentally ill parent, the child, the family, and the social environment (11). Protective factors can reduce the prevalence and/or severity of problems for COPMI (12). Supportive relationships, coping skills, positive relationships between parents, well-functioning communication within the family, and high socio-economic status are examples of such protective factors (13, 14). Several studies and meta-analysis have found significant effects of preventive interventions for COPMI (15–18). The results imply that preventive interventions with a psychoeducational focus reduce the risk for psychopathology and psychiatric symptoms and increase prosocial behavior for COPMI (15–18).

Knowledge about mental health provides resilience against mental illness (14, 19). Children who receive accurate, non-stigmatizing information about parental mental illness (PMI), treatment, and recovery may be able to understand their parent’s behavior, talk to others about their situation, and feel less alone (20). Knowledge and openness about PMI may reduce the stigma and burden of worrying for their parent and make it easier for children to seek professional help (14, 21). A lack of information about PMI can cause misunderstandings and misattributions of the causes of parent’s behaviors and treatment, and may increase feelings of concern, confusion, and stress for these children (14). In studies of which information COPMI value, children reported that they preferred to learn about PMI from healthcare professionals (HCPs) and regarded support and information as helpful (22, 23). They valued opportunities to ask questions and wanted to learn about the organization of health services. Several children wanted to be assured that it was not their fault that their parent was ill (22). Children expressed that they needed information about what a mental illness is, different types of illnesses, etiology and prognosis, how to cope with parents’ symptoms, where to seek help and support, and how to communicate with others about PMI (14).

Child Talks (CT) is a brief preventive intervention, developed in the Netherlands for COPMI aged 0–25 (24, 25). To this date there are no effect studies of the CT intervention. However, the CT has a clear and well-described theoretical foundation, focusing on psychoeducation. The intervention is delivered to patients with a mental illness and their children through three sessions. The patient’s child should be included in at least one session, to get information, ask questions and share any concerns. The intervention aims to strengthen parents’ knowledge of possible consequences for COPMI and increase parents’ focus on the child’s situation. By providing children with emotional and social support, and information about their parent’s disorder, treatment, and recovery, the intervention aims to reinforce children’s ability to cope with their situation. Another objective is to detect early signs of psychopathology and/or problem behaviors in children and initiate further support and referrals if needed. The intervention is manual-based and the sessions are described in detail in the CT manual (24). There is also a Logbook associated with the manual that HCPs should complete during or after CT sessions. The Logbook is described further in section “Child Talks Logbooks.”

The content of the CT intervention accords to §10 a) of the Health Personnel Act (26). The CT intervention was implemented in the participating clinic at the University Hospital of Northern Norway (UNN) when the amendments to the Health Personnel Act were made in 2010. The law states that HCPs are obligated to contribute to meet COPMIs need for information and support regarding their parents’ diagnoses and treatment. If necessary, HCPs should invite children to participate in a conversation to offer information and support. Despite the legal obligations, studies show that COPMI are not provided with the information they are entitled to (27, 28). Fewer than one third of HCPs had conversations with COPMI (28). Moreover, about 40% of parents in treatment reported that their children were unaware they were receiving treatment or being hospitalized, and over 40% reported that their children were not informed about their condition (27).

Most HCPs have positive attitudes toward a family-focused practice in adult mental health services (29). Still, studies have found numerous barriers for a family-focused practice (28–31). Important predictors for a family-focused practice are worker skills, knowledge, resources, and confidence, whereas families’ lack of time and fear of involving children are hindering factors (28, 29, 31, 32). Insecurities among HCPs about their role when meeting patients’ children and the lack of knowledge of how to have age-appropriate conversations about PMI with COPMI affected HCPs’ tendency to invite children negatively (33).

There is a lack of knowledge about how factors related to the parent and the child influence whether children are given information and support by the parent’s HCPs. Little is known about the extent to which COPMI participate in psychoeducational interventions and whether the children who do participate are provided with support, information, and follow-up actions. In this study we aimed to address this knowledge gap by analyzing patients’ health records.

The main aim of the present study was to evaluate the performance of CT sessions, with a particular focus on sessions with participating children. More specifically, we aimed to investigate:

(1) parents’ participation rate in CT,
(2) children’s participation rate in CT, and reasons for their exclusion,
(3) age, gender, and psychosocial differences between participating and non-participating children, and
(4) HCPs’ support and information to children.

## Materials and Methods

### Design

This is a retrospective study based on electronic patient journal data for the period 2010–2015. The approval from the data protection officer at UNN allowed us to extract the data from the electronic patient journals in 2015. The study has a mixed-methods approach since both quantitative data from electronic patient journal entries and written reports entered by HCPs to analyses qualitatively were used.

### Participants

The total HCPs workforce at the Division for Mental Health and Substance Use Disorders (DMHSD) at UNN was 436 in 2010 (29), whereas 35 HCPs held CT sessions with participating children. Family Assessment Forms were filled out for 424 patients.

### Data Material

The data material in this study is information extracted from the Family Assessment Forms and Logbooks from CT sessions, as recorded by HCPs in electronic patient journals. Over the course of the project, two different forms were implemented in the electronic patient journals at the DMHSD at UNN. These two forms were a Family Assessment Form and a Logbook from CT sessions. HCPs were instructed to fill out the Family Assessment Form for patients admitted to the DMHSD who had minor children. Secondly, the patients were to be invited to participate in CT, and HCPs were instructed to write a short report of the sessions in the electronic patient journal, labeled CT Logbooks. Information from these two forms was extracted from the electronic patient journals in 2015.

#### Family Assessment Forms

The Family Assessment Form consists of five categories of questions: (1) general information about the child, (2) the child’s network, (3) concerns for the child, and how the child is coping, (4) the child’s knowledge and information about PMI, and (5) the family’s need for support and follow-up.

#### Child Talks Logbooks

Logbooks from cases with participating children were used in the thematic analysis (*n* = 39). In the CT Logbooks, background information such as date, duration of session, place, participants, and parents’ diagnoses are requested. HCPs are also asked whether they have any concerns or issues regarding the family. In the following sections, HCPs are asked to respond to openly formulated questions about each session. Five sections are to be filled out for session one, two, and three about which topics and concerns were discussed, support options for the children and families, any questions regarding the child posed by the parents, any additional details, and agreements for the next session. For session three, there are additional sections for follow-up agreements and advice given to the child and parents, as well as for referrals and necessary follow-up actions that HCPs are to take. In our analyses, we used all the sections from sessions in which children participated. The amount of information and degree of details in the CT logbooks varied. In most logbooks HCPs had written a response in all sections. Some logbooks were several pages long, while others only contained a few paragraphs.

### Data Analyses

In the Family Assessment Forms each patient stated how many minor children they had. This information enabled us to calculate the total number of children for the assessed patients, and the number of children for the patients participating in CT. We detected how many children participated in CT from the Logbooks. Based on this information we calculated (1) children’s participation rate and (2) number of non-participating children whose parent participated. For non-participating children, we used information from the Logbooks to detect and quantify reasons for their absence.

Descriptive information of participating and non-participating children and parents was compared by analyzing information reported from CT sessions and Family Assessment Forms. For our analyses, we used information from the Family Assessment Form on parent gender, parent diagnosis, child age and gender, and where the children lived. We also used two questions about whether the children had received information about the parent’s treatment/hospitalization and condition: “*Does your child know that you receive treatment/are hospitalized?*” and “*Has your child received information about your condition?*” The response categories for these two items are “*no*”, “*partially*,” and “y*es*.” Descriptive statistics, *t*-tests, and chi-square testing were computed in IBM SPSS Statistics 25.

#### Statistical Analysis

We were not able to test differences between participating and non-participating children regarding parents’ diagnoses in reliable ways because of the small sample size, resulting in few parents in each diagnosis category. Partial receival of information was treated as having received information in the analysis. Chi-square tests was conducted to analyze differences between participating and non-participating children in terms of parent gender, child gender, information received and where the children normally lived. For all chi-square tests, we reported phi (φ) for effect size measurement. To test for differences in the mean age for participating and non-participating children, we initially performed a Levene’s test to determine if the variance of the groups was unequal or equal. The results from the Levene’s test showed that the variance of the groups was unequal. Therefore, we performed a two-tailed t-test with unequal variance for the groups to test for age differences. We calculated the effect size of the mean differences using Cohen’s *d*. The magnitude for all effect sizes was interpreted in accordance with Cohen (34).

#### Thematic Analysis

Child Talks Logbooks were analyzed to identify the thematic content of the sessions in which children participated, and all CT sessions with participating children were imported into the qualitative analysis program NVivo 12 Pro. The logbooks are a secondary source of information of the CT sessions, written and processed by HCPs.

For the thematic analysis of the CT sessions with participating children, the authors and researchers of the present study used the six-step phase guide by Braun and Clarke (35). This is a flexible approach in which the aim is to identify, analyze, and report the patterns found in the material (35). Our aim was to explore characteristics and patterns in the sessions with participating children; therefore, an inductive approach to the material was chosen.

The first step in the guide by Braun and Clarke (35) is getting to know the dataset by reading it multiple times. We transcribed the forms from paper to electronic format in order to familiarize ourselves with the material. Secondly, we started the initial coding of the material by identifying aspects of the data that reoccurred and which were an important focus in the sessions. The principle of data saturation was used, and hence we ended the initial coding when further coding no longer added new information. In the third step, we formed themes and sub-themes from the codes. In the fourth step, we reviewed all the themes and adjusted them as necessary so that the themes were more meaningful and comprehensive in respect of the codes included. In the fifth step, we defined the themes by writing a few sentences on their content that were suitable for all the codes included. The final, sixth step consisted of describing the themes in the present paper.

## Results

### Parents’ Participation Rate in Child Talks

Around 5,500 patients were receiving treatment at the DMHSD each year during the project period (2). A total of 424 patients were assessed as having minor children by using the Family Assessment Form, and 78 of these patients (18%) received the CT intervention.

### Children’s Participation Rate and Reasons for Exclusion

In 39 (50%) of the performed CT interventions all or some of patients’ children participated. The 78 patients who received the CT intervention had 157 children in total. Of these children, 62 (39%) took part in the intervention, leaving 95 children (61%) not participating despite their parent receiving the intervention.

**FIGURE 1:**
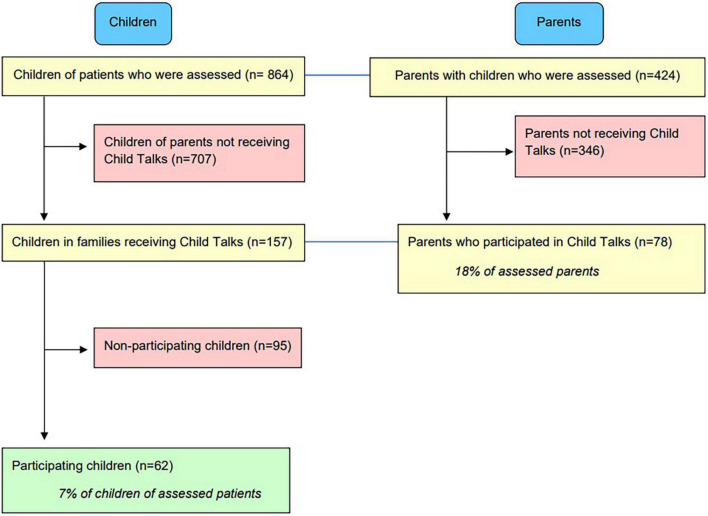
Childrens’ participation in Child Talks.

Based on the Family Assessment Forms, a total of 864 children were identified. Of these children, 62 participated in CT, resulting in a total participation rate of 7% for the identified children. See Figure 1 for a flowchart of childrens’ participation in CT.

Healthcare professionals provided information about the reasons for children’s absence in some of the records (*n* = 23). The reasons stated in records were: (1) the patient was soon to be discharged from hospital and therefore the task of talking to the children was postponed to a later occasion or transferred to personnel in other services(*n* = 7); (2) the patient had little contact with the child/children (*n* = 7); (3) the patient rejected the offer of CT with participating children (*n* = 5); and (4) the other parent of the child did not consent to the child participating, or personnel had not been given a response from the family (*n* = 4).

### Comparison of Participating and Non-participating Children

#### Diagnosis and Gender of Children’s Parents

The diagnosis and gender of participating and non-participating children’s parents are given in Table 1. Twelve parents had multiple diagnoses.

**TABLE 1:** Characteristics of parents with participating and non-participating children.

Characteristics of parents	With participating children (*n* = 39)	Without participating children (*n* = 39)
**Diagnosis**		
Alcohol and substance dependence	2	8
Paranoid schizophrenia and psychosis	7	1
Manic episodes	0	1
Bipolar disorder	5	4
Major depression disorder	15	17
Anxiety disorder	7	6
Post-Traumatic Stress Disorder	11	5
Eating disorder	1	1
Personality disorder	1	6
	49	49
**Patient’s kinship to children**		
Mother	33	26
Father	6	12
Sibling		1
	39	39

*Information missing on diagnoses of two parents with participating children. Since some parents had several diagnoses, the sum of diagnoses exceeds the number of patients participating in CT.*

The difference between participating and non-participating children in terms of parent’s gender was not significant at *p* < 0.05. The result from the chi-square test was *X*^2^ (1, *N* = 113) = 3.805, *p* = 0.051 and had a small to medium effect size (φ = 0.18). Information about parents’ gender was missing for 44 of parents’ children.

#### Childrens’ Age and Gender

**FIGURE 2:**
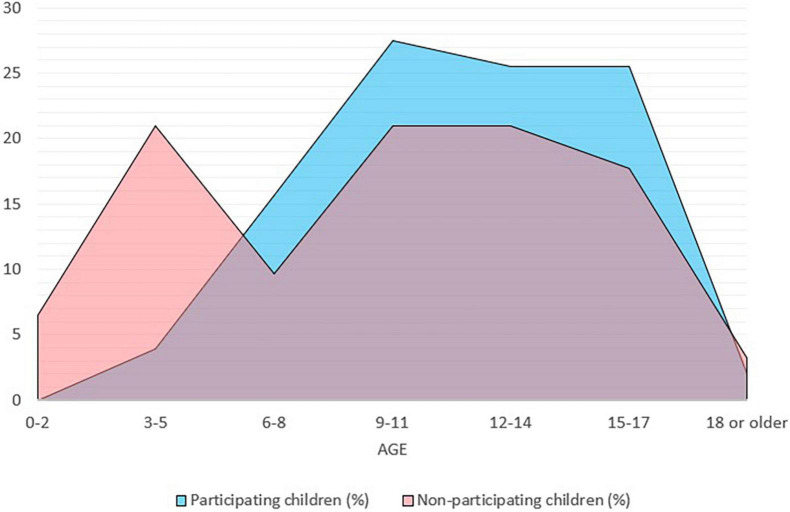
Age distribution for participating and non-participating children. *n* = 113, age missing for 11 participating children and 33 non-participating children.

Children participating in the intervention were between 3 and 22 years of age. We observed that the proportion of participating children increased with age (see Figure 2). Two children of preschool age (<6 years) participated (see Table 2). Of the participating children, 80% were more than nine years old. Figure 2 illustrates children’s age distribution for participating and non-participating children.

**TABLE 2:** Characteristics of participating and non-participating children.

Characteristics of children	Participating children (*n* = 62)	Non-participating children (*n* = 95)
**Gender**		
Girls	33	36
Boys	23	31
	56	67
**Age**		
0–2	0	4
3–5	2	13
6–8	8	6
9–11	14	13
12–14	13	13
16–18	13	11
18 or older	1	2
	51	62

*Information missing about gender for participating 6 participating and 28 non-participating children, and information missing about age for 11 participating children and 33 non-participating children.*

To test for difference in the mean of age of participating children [*M* (51) = 11.69, *SD* = 3.78] and non-participating children [*M* (62) = 10.13, *SD* = 5.27], we performed a *t*-test. The results from Levene’s test for difference of variance between the groups were significant at *p* < 0.05: *F* (1,111) = 5.764, *p* = 0.018. We therefore preformed a two-tailed t-test for which unequal variance for the groups was assumed. Cohen’s effect size value (*d* = 0.34) implied a small to medium magnitude of difference between the two groups, but the difference was not statistically significant at *p* < 0.05: *t* (111) = 1.83, *p* = 0.07.

Of the participating children, 33 were girls and 23 were boys. Of the non-participating children, 36 were girls and 31 were boys (see Table 2). Information about gender was missing for 6 participating and 28 non-participating children. A higher proportion of girls than boys participated. However, this difference was not statistically significant at *p* < 0.05, *X*^2^ (1, *N* = 123) = 0.335, *p* = 0.563 and had a very small effect size (φ = 0.05).

#### Information Provided to Children

Participating children knew that their parent was receiving treatment or was being hospitalized more often than children who did not participate in the sessions, measured at assessment point. Of the participating children, 97.7% (42 out of 43) were aware of their parent’s treatment or hospitalization, whereas 72.5% (29 out of 40) of the non-participating children were aware of this. However, answers to this question were missing for 19 participating children and 55 non-participating children. We compared participating and non-participating children by performing a chi-square test and found a significant difference of *p* < 0.05 between the groups, *X*^2^ (1, *N* = 83) = 10.62, *p* = 0.001, with a medium magnitude of difference (φ = 0.36).

Participating children had also received information about their parent’s condition more often than non-participating children, at assessment point. Of the participating children, 85.7% (36 out of 42) were aware of their parent’s condition, compared to 60.5% (23 out of 38) of the non-participating children. Answers to this question were missing for 20 participating and 57 non-participating children. The results from the chi-square test comparing the two groups showed a significant difference at *p* < 0.05 level, *X*^2^ (1, 80) = 6.54, *p* = 0.011, with a small-to-medium effect size (φ = 0.28).

#### Where the Children Lived

For participating children, 94% (47 out of 50) lived with the hospitalized parent. In the case of the non-participating children, 65.9% (29 out of 44) were registered as living with the patient. Answers to the question of where the children lived were missing for 12 out of 62 participating children and 51 out of 95 non-participating children. A chi-square test conducted to assess the difference between participating and non-participating children in terms of living with the hospitalized parent showed a significant difference at *p* < 0.05, *X*^2^ (1, *N* = 94) = 11.93, *p* = 0.001, with a medium effect size (φ = 0.36).

### Themes in Sessions With Participating Children

**FIGURE 3:**
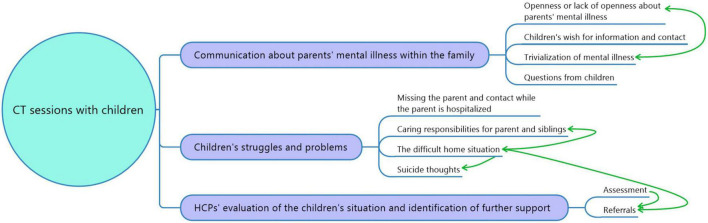
Map over main themes and sub themes.

The thematic analysis of the written reports from CT sessions involving children resulted in three main themes and ten sub-themes. The main themes were communication about PMI within the family, children’s struggles, as well as HCPs’ evaluation of the child’s situation and identification of further support. See Figure 3 for an overview of main themes and sub-themes.

#### Informing Children About Parental Mental Illness

Healthcare professionals frequently explained to parents why talking openly about mental health within the family is important. Some of the parents were open about their illness, and many said they wanted to be even more open. In many families, however, there was little to no communication about the parent’s illness. Hence, for some children the CT session was the first time they received information about their parent’s illness. The children usually expressed that they were glad to receive information from HCPs. However, some children did not want information and did not want to visit or have contact with the mental health services.

In some cases, the parents were unwilling to talk about mental illness and prevented their children from receiving information or being given the opportunity to talk about their situation. Reasons were that they did not want their children to be unnecessarily worried or to focus on the negative characteristics and psychiatric diagnosis of the sick parent. A mother who was diagnosed with depression, anxiety, and an eating disorder protected her 10-year-old daughter (ID 35), from words such as “mental illness” and “psychiatric hospital.”

The information that children received was not always correct or clarifying. Proper explanations about parents’ illness, symptoms and behavior were often missing. Some children knew that something was wrong or that their parents were struggling, but not what or how. Often parents and HCPs used word such as “exhausted” or “having headache” when explaining parents’ mental illness.

Questions from children appeared in a few records. The questions reported concerned the care of younger siblings, heredity, and the home situation. Children did not usually ask directly, but rather expressed their curiosity about a theme in the session.

#### Children’s Struggles and Problems

Children reported missing their hospitalized parents and being worried about them. Two boys aged 10 and 11 years (ID 78) missed their father while he was hospitalized and reported being worried about him. This was also given as the reason for the youngest boy having trouble concentrating at school. Some children kept in contact with the hospitalized parent by using video calls and some had also visited their hospitalized parent on several occasions. Some children missed the way things used to be before their parent became ill, like a 15-year-old girl (ID 17) who said that she was missing her “healthy” mother. She missed spending time with her mother in the evenings, lighting candles, and watching movies. She also missed tasty and healthy meals.

Several children in the records were worried about, and had great responsibilities for, the care of their sick parent and/or younger siblings. A 12-year-old girl (ID 42) had to physically stop her mother from dying by suicide. The girl was worried about what might happen once her mother was discharged from the hospital. HCPs emphasized the importance of making the daughter aware that it was not her responsibility to take care of and look after her mother, yet they advised her to contact the police if her mother did anything like that again. The girl also expressed her concerns for the care of her younger siblings while her mother was hospitalized, especially the one-year-old. She was not sure her father would be able to take care them. She had trouble sleeping at night. The girl was advised to contact help services if things became difficult or if she needed someone to talk to.

Some children were overinvolved in the illness of their parent, such as a 12-year-old boy (ID 18) whose mother had been diagnosed with anxiety:

*The boy said he was going to look after his mother until he became an adult. He was worried about his mother when she got her anxiety attacks. When she got the anxiety attacks, he massaged her.*

Another child, a 16-year-old boy (ID 60), frequently had to participate in his mother’s doctor appointments and translate letters from the Ministry of Foreign Affairs. He was also involved in the conflict between his parents and described how he had to stop his father from being violent with his mother:

*In the beginning, after they came to Norway, his father was physically violent toward his mother. One night he got up and told his father that if he ever beat her again, he would call the police. The father went out and did not return until much later. Since the incident the father has never beaten her. The boy cried while telling this. He said this was the very first time he had cried in front of his sister.*

Many children had a difficult home situation, living in families with severe, long-lasting problems. Many of the children had experienced frightening episodes at home. The children’s situations at home were often described as unpredictable, stressful, and characterized by high conflicts levels and violence between family members. In one case a 16-year-old girl (ID 39) who was living with her mother, who had been diagnosed with a psychotic illness, was physically abused:

*There was general concern for the family because of the mother’s mental state. There was also concern for the daughter’s situation and whether she was given help for her own mental health problems, which she had had for several years. Her mother was unstable and had on several occasions pushed and thrown things after the girl. The mother had also called her names. It was difficult for the daughter because her mother was suspicious and seemed to be in a paranoid state of mind.*

The patient in the example above was discharged from the hospital and sent home to her daughter. A report of concern was sent to The Child Welfare and Protection Services (CWPS). The daughter was advised to contact the school nurse when she returned to school at the end of the summer.

Several children had more or less concrete thoughts about dying by suicide. For one of these children, HCPs stated that the child’s mental health problem was taken care of by their general practitioner. In another case a 16-year-old boy (ID 60) was invited to call HCPs if he wanted to talk, after he had told them that he had thought about cutting his main artery if he was sent back to Afghanistan. He had even looked in the kitchen drawer for a knife. For a child who had attempted to die by suicide earlier, HCPs were concerned for the child’s mental health problems and whether appropriate help was provided.

#### Evaluating and Supporting the Children

In many of the records, HCPs observed and evaluated the situation of the children and the parent’s caring abilities. In some cases, HCPs explicitly wrote down the agenda for evaluating the children, for example to “look at the interaction between the mother and the daughter” or to “observe the relation and the interaction between the children and parents.” In one case (ID 26), the HCPs evaluated the attachment and how the child acted around his parents. HCPs even talked with the four-year-old boy while his parents were waiting outside.

Healthcare professionals explored the children’s network and support options as part of the intervention. Many sessions led to referrals to CWPS and The Child and Adolescent Mental Health Services (CAMHS), but in some cases where children described severe problems, no referrals were made. HCPs frequently encouraged parents and children to contact their general practitioner, their teacher, or the school nurse. Responsibility for establishing contact with help services or professionals was often left with the child. Some families were already in contact with CWPS and/or CAMHS. School nurses were frequently recommended as a support option. In the case of the 10-year-old girl (ID 35) who was being shielded from words such as “mental illness” and “psychiatric hospital,” the school nurse was recommended as a support option because the parent and the child knew of her. Likewise, in a case with a 17-year-old boy (ID 09), the school nurse was emphasized as a support option as a neutral person the boy could talk to about everyday life and other relevant topics of conversation for adolescents. In addition, HCPs often gave children and parents the opportunity to have several sessions and to get in touch with them outside of the sessions if they needed to talk. The HCPs also gave children the opportunity to call them if they had any questions. In some cases, there was a mutual agreement that the family would benefit from staying in touch with the ward.

## Discussion

### Is the Participation Rate of Patients at an Acceptable Level?

Of the thousands of patients at the DMHSD at UNN during the period 2010–2015 (2), only 424 were assessed with the Family Assessment Form. This means that for most of the patients, there were no records of minor children they might be parenting. Furthermore, only 78 patients received the CT intervention, meaning that only a small fraction of COPMI were attended to in the manner mandated by legislation in Norway. These results are in line with previous research, suggesting that it is challenging to implement new routines related to COPMI in Norway (36, 37) and illustrating the need for a better implementation strategy.

### Is the Participation Rate of Children at an Acceptable Level?

Seven percent of children identified in the Family Assessment Forms participated in CT. However, the number of children identified in the Family Assessment Form does not represent all minor children of patients at the DMHSD. In fact, around 5,500 patients were receiving treatment at the DMHSD each year during the project period (2), and likely one third of them had a mean of 1.75 minor children each (3, 27, 38), which equals more than three thousand minor children of patients each year. The participation rate based on the actual number of minor children of patients will therefore be considerably lower than 7%.

According to the CT manual, children should participate in the second session and optionally in the third session (24). However, children were only present in half of the cases in which the CT intervention was utilized, and since not all siblings participated in cases including children, children’s participation rate in received CT was only 39%. Children’s participation rate reveals that the intervention manual is not being adhered to, and consequently, the obligation of HCPs to provide COPMI necessary information about PMI, support and follow up does not seem fulfilled. It is especially important that HCPs provide information to COPMI since previous research has shown that parents themselves often did not inform their child about their treatment/hospitalization or condition (27).

The main reasons for children not participating reported in this study were reluctance of one or both parents, little contact with the children or ending of the parent’s treatment. In cases where the parent does not have custody or contact with the children, inviting the children to participate in a session is not appropriate, thus these patients would not have been invited to participate in the CT intervention. Large demographical distances between the clinic and the childrens home might have made childrens participation difficult in some cases, especially for the youngest children. In cases in which parents were reluctant to bring their children to a CT session, HCPs are in a good position to argue in favor of child participation. The reasoning behind including children is available in the CT manual and motivating parents and planning how to inform the children is the core activity in session one (24). Aligned with previous research, HCPs seem to need better awareness of the importance of giving children information and support, greater skill at motivating parents to invite their children, and greater skill, or perhaps greater confidence, in performing conversations with children present (28, 31).

### Which Factors Influence Child Participation?

In terms of factors relating to the parent, differences between participating and non-participating children regarding parents’ diagnoses could not be tested in reliable ways because of the sample size. However, the parent’s gender might be a factor influencing child participation, with a difference between participating and non-participating children close to our chosen significant level, with a small to medium effect size. COPMI more often lived with their mothers as a sole caregiver and therefore were in the care of relatives while their mother was hospitalized (27). A more dramatic change in these children’s life situations calls for more information and support, which might explain why children of mothers participated more often. This is also in correspondence with our results showing that participating children more often lived with the parent in treatment. Furthermore, deciding and planning for child participation is easier when the child is fully under the custody of the patient.

Mostly older children participated in CT. Child participation increased with child age, and the difference between participating and non-participating children in mean age was close to our chosen significance level, with a small to medium effect size. In earlier studies HCPs have reported feeling insecure about who has the responsibility of children visiting patients and how to have age-appropriate conversations (33). Furthermore, the study found that HCPs’ confidence level influenced their initiative to motivate patients to invite their children. For the youngest children, it is possible that HCPs’ insecurities were amplified, since younger children are less independent and require more adjustments by HCP. HCPs might need more knowledge and training in child development and age-appropriate conversations about PMI. Interventions for COPMI can also be more adaptable and user-friendly for HCPs, by making recommendations for different age groups. For example, for children under two years a visit to the hospital to assure them their parent is safe can be recommended. For children from three years and up, in addition to recommending a visit, guidelines for age-appropriate information and communication principles can be provided in the intervention manual. Increasing HCPs’ information and support to the youngest children is of great importance since younger children are the most dependent on their parents, and not mature and autonomous to seek information and support elsewhere. We found no differences in child gender for participation.

In terms of knowledge about PMI, there was a significant difference, of a small to medium magnitude, between participating and non-participating children. Participating children more often already knew about parents’ treatment/hospitalization and condition. Families that are more open about PMI might be more willing to have children participating in a conversation with HCPs. This is in coherence with earlier studies in which families’ fear of involving children was perceived as an important hindering factor for a family-focused practice by HCPs (32).

### Are Children Supported and Informed?

The main themes in the CT sessions with children reflected the objectives of the CT intervention (24): communication about PMI within the family, children’s struggles and HCPs’ evaluation of the child’s situation and identification of further support. However, the content of the CT sessions uncovered a large variation in the quality of the support and information children were provided.

Children were glad to receive information, which is in line with earlier studies which show that children appreciate support and information from HCPs (22, 23). Parents, however, were sometimes reluctant and unsure about informing their children because they did not want to make their child additionally worried, a barrier for family-focused practice found in another study (32). Our result confirmed, what is described by other researchers (14), that children often know that something was wrong and that not having information could lead to frustration. It was also found that the children were missing their parent and were worried about them. These results underpin the importance of information and contact with the parent in treatment for COPMI (14, 19–22).

Healthcare professionals evaluated and explored children’s situations but were reluctant to refer to other services or provide further support. High conflict levels within the family, domestic violence, physical and mental abuse, mental health problems and suicide thoughts among children were described. However, few appropriate actions were taken by HCPs. Despite HCPs’ obligation by law to refer children to the CWPS when concerned with their living situation, HCPs did not take appropriate actions in all cases. In previous studies HCPs have reported hesitation against referrals because of insecurities of whether there were grounds for referral, whether a referral would benefit the child and whether a referral would harm the family and their relationship with the patient (39). The lack of action by HCPs does not only take away children’s chance for help but does also trivialize the problems and struggles the children are experiencing. HCPs need to know which support they can offer, and which actions to take. Educating HCPs about follow-up options and help services for children might contribute to providing COPMI better support.

### Strengths and Limitations

One limitation of this study is missing data for several variables, particularly for the non-participating group of children. The results must therefore be interpreted carefully. The small sample size may be a factor contributing to the lower sensitivity of the t-test, resulting in less reliable results.

The journal data were a secondary source of information of the CT sessions, written and processed by HCPs, based on their perception of what is important and of interest. What was written in the logbooks was partially decided and influenced by the CT logbook and the questions HCPs were to answer. However, the questions were openly formulated and did invite HCPs to give detailed descriptions of the conversations and share a range of information. Despite this, it varied how much and how specific the written information about the conversations were. By focusing on the themes that were discussed in sessions, rather than looking for meanings behind the text, the data material was suitable to answer the associated research aim in the present study. The benefit of the research design is that it enabled a reduction of the disturbance and influence of an observing researcher. A researcher present in sessions might have made participants more hesitant to speak openly about sensitive and personal subjects. In addition, HCPs should be able to perform the sessions at a time they found appropriate in respect of the patient’s course of treatment and time management. Having to plan for a third person’s participation would have made the feasibility of the project weaker.

Only CT Logbooks in the electronic patient journal were assessed; hence information written elsewhere was not available and not included in the analysis. What is logged from the sessions is partially prearranged from the Logbook forms. Since the Logbook forms are directly based on the manual’s description of the intervention, the data may incorrectly confirm the HCPs’ adherence to the manual. The HCPs were aware that the reports were going to be used in a quality-assurance project and they might therefore have reported the session more in line with the guidelines of the manual. There was, however, sections in the Logbook form with open formulated questions, which gave HCPs the opportunity to share a wide range of information.

### Future Research

To facilitate and strengthen the degree to which children are given information and support they are entitled, more research is needed to gain detailed knowledge about factors influencing children’s participation. Future research should identify reasons why HCPs are not including children and investigate whether it is due to lack of consent from parents, institutional constraints, or unfulfilled professional needs. It would be useful to know whether certain characteristics of the parent’s illness, such as a sudden onset or a significant change in the parent’s functional level and behavior, influence the need to give and receive information among HCPs, parents, and children. Whether child participation is influenced by parent gender also needs to be explored in future studies after adjusting for where the child usually lives. In addition, the difference between participating and non-participating children in terms of received information about PMI should be investigated when adjusting for confounding factors, such as the child age.

## Conclusion

Child Talks is an intervention that seeks to reinforce COPMI’s ability to cope with their family situation by the provision of age-appropriate information about their parent’s illness and treatment. The intervention also aims to provide additional support and follow-up for the children who require it. Of patients who were registered as having minor children, less than one fifth received the intervention, and only half of the patients who participated also had their children participating. Of the registered minor children, less than one in ten received CT. Ideally, children who participate in the intervention emerge better informed, supported and are, when necessary, provided with follow-up. However, this study shows that even participating children were not always followed-up or judged to have been adequately informed. Routines and training of HCPs to support parents with mental illness and their children need improvement. Initial identification of children of patients is important, and subsequent support and provision of adequate services to the identified children always needs to follow.

## Data Availability Statement

The original contributions presented in the study are included in the article/supplementary material, further inquiries can be directed to the corresponding author.

## Ethics Statement

The studies involving human participants were reviewed and approved by the Data Protection Officer at the University Hospital of Northern Norway (UNN). The regional ethics committee (REK) categorized the project as a quality assurance project. Written informed consent from the participants’ legal guardian/next of kin was not required to participate in this study in accordance with the national legislation and the institutional requirements.

## Author Contributions

KK made the analysis and the draft of the manuscript. CR was the primary investigator in the main project. CR and CL collected the data in collaboration with the project coordinator, Lisbeth Mørch, at UNN. All authors contributed to the writing of the manuscript.

## Conflict of Interest

The authors declare that the research was conducted in the absence of any commercial or financial relationships that could be construed as a potential conflict of interest.

## Publisher’s Note

All claims expressed in this article are solely those of the authors and do not necessarily represent those of their affiliated organizations, or those of the publisher, the editors and the reviewers. Any product that may be evaluated in this article, or claim that may be made by its manufacturer, is not guaranteed or endorsed by the publisher.

## Abbreviations

CAMHS: The Child and Adolescent Mental Health Services
COPMI: Children of parents with a mental illness
CT: Child Talks
CWPS: The Child Welfare and Protection Services
DMHSD: The Division for Mental Health and Substance Use Disorders
HCPs: healthcare professionals
PMI: Parental mental illness.

## References

[1] Torvik FA Rognmo K. *Barn av Foreldre med Psykiske Lidelser eller Alkoholmisbruk: Omfang og Konsekvenser.* Oslo: Nasjonalt folkehelseinstitutt (2011).

[2] Reedtz C Mørch L Lauritzen C. Registreres psykiatriske pasienters barn i elektronisk pasientjournal? *Nordisk Sygeplejeforskning.* (2015) 5:36–45.

[3] Maybery D Reupert AE. The number of parents who are patients attending adult psychiatric services. *Curr Opin Psychiatry.* (2018) 31:358–62. 10.1097/YCO.0000000000000427 29847344

[4] Reupert AE Maybery D Kowalenko N. Children whose parents have a mental illness: prevalence, need and treatment. *Med J Aust.* (2013) 199:S7–9. 10.5694/mja11.11200 25369850

[5] Weissman M Wickramaratne P Gameroff M Warner V Pilowsky D Kohad R Offspring of depressed parents: 30 years later. *Am J Psychiatry.* (2016) 173:1024–32. 10.1176/appi.ajp.2016.15101327 27113122

[6] Sundfær A. *God Dag, Jeg er et Barn – Om Barn som Lever Med rus Eller Psykisk Sykdom i Familien [Good Day, I am a Child – About Children Living With Substance use or Mental Illness Within Their Family].* Oslo: Fagbokforlaget (2012).

[7] Brennan PA Hammen C Andersen MJ Bor W Najman JM Williams GM. Chronicity, severity, and timing of maternal depressive symptoms: relationships with child outcomes at age 5. *Dev Psychol.* (2000) 36:759–66. 10.1037//0012-1649.36.6.759 11081699

[8] Farahati F Marcotte DE Wilcox-Gök V. The effects of parents’ psychiatric disorders on children’s high school dropout. *Econ Educ Rev.* (2003) 22:167–78.

[9] van Santvoort F Hosman CMH Janssens JMAM van Doesum KTM Reupert A van Loon LMA. The impact of various parental mental disorders on children’s diagnoses: a systematic review. *Clin Child Fam Psychol Rev.* (2015) 18:281–99. 10.1007/s10567-015-0191-9 26445808

[10] Rasic D Hajek T Alda M Uher R. Risk of mental illness in offspring of parents with schizophrenia, bipolar disorder, and major depressive disorder: a meta-analysis of family high-risk studies. *Schizophr Bull.* (2014) 40:28–38. 10.1093/schbul/sbt114 23960245 PMC3885302

[11] Hosman CMH van Doesum KTM van Santvoort F. Prevention of emotional problems and psychiatric risks in children of parents with a mental illness in the Netherlands: I. The scientific basis to a comprehensive approach. *Adv Ment Health.* (2009) 8:250–63. 10.5172/jamh.8.3.250

[12] Foster K O’Brien L McAllister M. Addressing the needs of children of parents with a mental illness: current approaches. *Contemp Nurse.* (2004) 18:67–80. 10.5172/conu.18.1-2.67 15729799

[13] Foster K O’Brien L Korhonen T. Developing resilient children and families when parents have mental illness: a family-focused approach. *Int J Ment Health Nurs.* (2011) 21:3–11. 10.1111/j.1447-0349.2011.00754.x 21692961

[14] Riebschleger J Grove C Cavanaugh D Costello S. Mental health literacy content for children of parents with a mental illness: thematic analysis of a literature review. *Brain Sci.* (2017) 7:141. 10.3390/brainsci7110141 29072587 PMC5704148

[15] Siegenthaler E Munder T Egger M. Effect of preventive interventions in mentally ill parents on the mental health of the offspring: systematic review and meta-analysis. *J Am Acad Child Adolesc Psychiatry.* (2012) 51:8–17.e8. 10.1016/j.jaac.2011.10.018 22176935

[16] Solantaus T Paavonen EJ Toikka S Punamäki R-L. Preventive interventions in families with parental depression: children’s psychosocial symptoms and prosocial behaviour. *Eur Child Adolesc Psychiatry.* (2010) 19:883–92. 10.1007/s00787-010-0135-3 20890622 PMC2988995

[17] Thanhäuser M Lemmer G de Girolamo G Christiansen H. Do preventive interventions for children of mentally ill parents work? Results of a systematic review and meta-analysis. *Curr Opin Psychiatry.* (2017) 30:283–99. 10.1097/YCO.0000000000000342 28505032

[18] Lannes A Bui E Arnaud C Raynaud JP Revet A. Preventive interventions in offspring of parents with mental illness: a systematic review and meta-analysis of randomized controlled trials. *Psychol Med.* (2021) 51:2321–36. 10.1017/S0033291721003366 34435556

[19] Reupert AE Maybery D. “Knowledge is power”: educating children about their parent’s mental illness. *Soc Work Health Care.* (2010) 49:630–46. 10.1080/00981380903364791 20711943

[20] Grové C Melrose H Reupert A Maybery D Morgan B. When your parent has a mental illness: children’s experiences of a psycho-educational intervention. *Adv Ment Health.* (2015) 13:127–38.

[21] Pihkala H Sandlund M Cederstrom A. Children in Beardslee’s family intervention: relieved by understanding of parental mental illness. *Int J Soc Psychiatry.* (2012) 58:623–8. 10.1177/0020764011419055 21900288

[22] Grové C Reupert A Maybery D. The perspectives of young people of parents with a mental illness regarding preferred interventions and supports. *J Child Fam Stud.* (2016) 25:3056–65.

[23] Drost LM van der Krieke L Sytema S Schippers GM. Self-expressed strengths and resources of children of parents with a mental illness: a systematic review. *Int J Ment Health Nurs.* (2016) 25:102–15. 10.1111/inm.12176 26692281

[24] Reedtz C Lauritzen C. *Håndbok – Prosjekt Barn som Pårørende. Regionalt Kunnskapssenter for Barn og Unge.* Oslo: RKBU Nord (2011).

[25] Doesum KTV Koster C. *KOPP Praten met Ouders Kinderen: Handleiding Preventieve Huisbezoeken voor Ouders met Psychiatrishe Problemen en Hun Kinderen Manual Child Talks.* (2008). Deventer, The Netherlands: Dimence, Community Mental Health Center.

[26] The Health Personnel Act, §10 a (2010). *Act Relating to Health Personnel (LOV 1999-07-02-64)*. Lovdata. Available online at: https://lovdata.no/dokument/NL/lov/1999-07-02-64/KAPITTEL_2#%C2%A710a

[27] Reedtz C Lauritzen C Stover YV Freili JL Rognmo K. Identification of children of parents with mental illness: a necessity to provide relevant support. *Front Psychiatry.* (2019) 9:728. 10.3389/fpsyt.2018.00728 30670987 PMC6333019

[28] Skogøy BE Ogden T Weimand B Ruud T Sørgaard K Maybery D. Predictors of family focused practice: organisation, profession, or the role as child responsible personnel? *BMC Health Serv Res.* (2019) 19:793. 10.1186/s12913-019-4553-8 31684933 PMC6829823

[29] Lauritzen C Reedtz C Van Doesum K Martinussen M. Factors that may facilitate or hinder a family-focus in the treatment of parents with a mental illness. *J Child Fam Stud.* (2015) 24:864–71. 10.1007/s10826-013-9895-y 25814823 PMC4363479

[30] Maybery D Reupert A. Parental mental illness: a review of barriers and issues for working with families and children. *J Psychiatr Ment Health Nurs.* (2009) 16:784–91. 10.1111/j.1365-2850.2009.01456.x 19824972

[31] Maybery D Goodyear M Reupert AE Grant A. Worker, workplace or families: what influences family focused practices in adult mental health? *J Psychiatr Ment Health Nurs.* (2016) 23:163–71. 10.1111/jpm.12294 27170070

[32] Korhonen T Vehviläinen-Julkunen K Pietilä A-M. Implementing child-focused family nursing into routine adult psychiatric practice: hindering factors evaluated by nurses. *J Clin Nurs.* (2008) 17:499–508. 10.1111/j.1365-2702.2007.02008.x 18205682

[33] O’Brien L Brady P Anand M Gillies D. Children of parents with a mental illness visiting psychiatric facilities: perceptions of staff. *Int J Ment Health Nurs.* (2011) 20:358–63. 10.1111/j.1447-0349.2011.00740.x 21385296

[34] Cohen J. *Statistical Power Analysis for the Behavioral Sciences.* Mahwah, NJ: Lawrence Erlbaum Associates (1988).

[35] Braun V Clarke V. Using thematic analysis in psychology. *Qual Res Psychol.* (2006) 3:77–101.

[36] Reedtz C Jensaas E Storjord T Kristensen KB Lauritzen C. Identification of children of mentally Ill patients and provision of support according to the Norwegian health legislation: a 11-year review. *Front Psychiatry.* (2022) 12:815526. 10.3389/fpsyt.2021.815526 35095621 PMC8795076

[37] Lauritzen C Reedtz C Van Doesum KTM Martinussen M. Implementing new routines in adult mental health care to identify and support children of mentally ill parents. *BMC Health Serv Res.* (2014) 14:58. 10.1186/1472-6963-14-58 24507566 PMC4015279

[38] Statistics Norway. *Slik er Norske Barnefamilier [How Norwegian Families with Children Are].* (2018). Available online at: https://www.ssb.no/befolkning/artikler-og-publikasjoner/slik-er-norske-barnefamilier (accessed May 15, 2021).

[39] Lauritzen C Reedtz C. Adult mental health services and the collaboration with child protection services. *J Hosp Admin.* (2016) 5. 10.5430/jha.v5n5p72

